# Editorial: Microbiome-driven strategies for alleviation of biotic and abiotic stresses in agriculture

**DOI:** 10.3389/fmicb.2026.2004577

**Published:** 2026-10-13

**Authors:** Satish Kumar, Kamlesh K. Meena, Ajay M. Sorty, Goraksha C. Wakchaure, Mahesh Kumar

**Affiliations:** 1 ICAR-Directorate of Onion and Garlic Research, Pune, MH, India; 2 ICAR-Central Arid Zone Research Institute, Jodhpur, Rajasthan, India; 3 Aarhus University, Roskilde, Denmark; 4 ICAR- National Institute of Abiotic Stress Management, Baramati/Pune, MH, India; 5 ICAR-Indian Agricultural Research Institute, New Delhi, India

**Keywords:** abiotic stress, biotic stress, disease suppression, microbiome engineering, plant microbiome, rhizosphere

Over recent decades, advances in plant microbiome research have transformed our understanding of plants as complex biological systems integrated with diverse microbial communities, whose interactions shape the functioning and fitness of the plant holobiont (Berg et al., 2016). This conceptual shift is reinforced by evidence that plant-associated microbiomes are shaped by host, environmental, and management factors, including biotic and abiotic stresses, and, in turn, influence plant performance through nutrient acquisition, phytohormone regulation, pathogen suppression, stress signaling, and host physiological responses (Trivedi et al., 2020). Building on these advances, plant–microbe research is moving beyond taxonomic profiling toward understanding microbiome assembly and function, while exploring synthetic communities and targeted microbiome manipulation for predictable, persistent field benefits. In this context, this Research Topic brings together 11 contributions that provide complementary perspectives on how plant-associated microbial communities respond to biotic and abiotic stresses, how their assembly and functions are shaped by the host and its environment, and how a deeper understanding of these interactions may help harness beneficial microbiome functions for crop resilience.

Among the themes explored in this Research Topic, the relationship between resident microbial communities and plant disease offers compelling evidence of the plant microbiome's functional importance. The capacity of indigenous microbiota to suppress pathogen establishment and disease development was recognized through the phenomenon of disease-suppressive soils (Cook and Rovira, 1976), with subsequent community-level analyses identifying microbial taxa and interactions associated with such suppression (Mendes et al., 2011). Extending this concept, Jobert et al. demonstrated that plant health status in rice can leave a persistent rhizosphere microbial legacy influencing growth and disease response in the subsequent generation. Rice grown in rhizosphere mixes derived from diseased plants (DRM) exhibited 30% shorter shoots but developed 44% shorter lesions following challenge with *Xanthomonas oryzae pv. oryzae* (Xoo), compared with plants grown in healthy rhizosphere mixes (HRM). These findings are consistent with the “cry for help” framework, whereby pathogen-stressed plants may recruit protective microbiota (Rolfe et al., 2019), potentially contributing to persistent disease-suppressive microbial states.

Beyond plant–soil feedbacks, understanding microbial sources and assembly across cropping seasons and plant development is crucial to explaining disease-associated microbiome dynamics. Whitaker et al. examined the spatial and temporal dynamics of microbiome assembly in the wheat–Fusarium head blight (FHB) pathosystem by tracking fungal communities in maize debris, wheat leaves, and heads across five developmental stages, from pre-winter tillering to dough filling, at two locations. Microbial source tracking revealed that preceding-season maize debris contributed approximately 70% of the early wheat-leaf mycobiome, whereas wheat leaves contributed only a small proportion to wheat-head fungal communities. Together, these findings suggest that disease-associated microbiome states reflect not only pathogen pressure but also microbial sources, developmental timing, and tissue-specific community assembly. Beyond these factors, host genotype influences microbial community composition and development across successive cropping cycles. Xiang et al. investigated corm-associated bacterial communities in banana, where successive crops arise from suckers connected to the mother plant through the underground corm, potentially enabling endophyte persistence and transfer. Across ratoon cycles, resistant cultivars developed increasingly distinct and diverse corm bacterial communities, enriched in *Halomonas* and *Nesterenkonia*, alongside a decline in Fusarium wilt incidence from 34–39% to 4–8%. Complementing these genotype-associated microbiome patterns, Zhang and Li combined rhizosphere community profiling and culture-based screening across six alfalfa cultivars differing in Verticillium wilt resistance. Comparing rhizosphere bacterial communities, they found that the highly resistant cultivar “*Gannong No. 4*” was enriched in *Devosia* and *Novosphingobium* relative to susceptible cultivar “*Saranac*.” Together, these studies highlight how microbial legacies, colonization history, tissue specificity, and host genotype shape disease-associated microbiome assembly. Beyond community-level dynamics, Fu et al. revealed distinct transcriptomic and metabolomic responses of *Ustilaginoidea virens* during infection of indica and japonica rice, highlighting pathogen-level processes that complement our understanding of host genotype-dependent disease interactions.

Abiotic stresses add another dimension to microbiome-mediated plant resilience by imposing environmental selection pressures that reshape microbial community assembly, functional potential, and metabolic processes associated with host stress responses. Liu et al. used integrated shotgun metagenomics and untargeted metabolomics to characterize the rhizosphere response of *Praxelis clematidea* to 5°C vs. 25°C. Although alpha diversity changed little, cold stress altered community composition, enriching *Nocardiopsis, Sphingobium, Amycolatopsis*, and *Azoarcus*, and reorganized genes related to carbon and nitrogen metabolism, with greater representation of glycolytic and TCA-cycle functions but reduced abundance of nitrogen-assimilation genes such as *glnA* and *nasA*. Furthermore, agronomic management practices can indirectly steer rhizosphere microbial assembly by modifying soil physicochemical conditions and resource availability. Fan et al. investigated whether modifying the physical form of wheat straw could influence microbial community organization and carbon cycling under salinity and temperature constraints. In a 360-day soil incubation experiment, they compared equal-carbon inputs of chopped straw, straw-derived biochar, and granular straw across salinity and temperature gradients. Granular straw increased fungal richness relative to chopped straw and distinctly restructured microbial communities, with greater complexity and connectivity in bacterial and fungal co-occurrence networks. These changes were associated with soil organic carbon fractions, extracellular enzyme activities, and carbon mineralization, highlighting how residue formulation can influence microbial assembly and carbon turnover in saline soils. At the individual microbe–host level, Hua et al. provided a complementary mechanistic perspective using the soybean endophyte *Priestia megaterium* 170T-4. Under salinity stress, inoculation improved plant growth and Na^+^/K^+^ homeostasis, while transcriptomic analysis indicated modulation of ion-transport genes and MAPK, ethylene, and jasmonate signaling pathways. Beyond vegetative tissues, microbial assembly in reproductive organs is also shaped by interactions between plants and their biotic environment. Li et al. addressed the poorly understood contribution of insect visitation to pollen microbiome assembly by comparing open and insect-excluded flowers across four crops with contrasting pollination strategies. Host identity and insect visitation differentially structured bacterial and fungal communities, with fungi showing greater compositional sensitivity.

Collectively, these contributions advance ecological and functional understanding of plant microbiomes while revealing key gaps. Amplicon-based profiling still predominates, underscoring the need for greater integration of shotgun metagenomics and functional multi-omics to resolve underlying mechanisms. Hibatullah et al. similarly emphasized integrating multi-omics-guided rhizobiome engineering with regenerative soil management, while highlighting the need for long-term field validation. Crucially, associations between microbial taxa and biotic or abiotic stress resistance do not establish causality, nor do co-occurrence networks confirm direct microbial interactions. Zhao et al. highlighted microbiome steering and synthetic communities as emerging strategies, while emphasizing the need for stable establishment and field validation. Longitudinal studies across host genotypes, cropping cycles, environments, and management regimes are needed to establish the stability and context dependence of microbiome functions. Cross-kingdom interactions among bacteria, fungi, archaea, protists, and viruses remain underexplored, particularly their collective roles in pathogen suppression, nutrient cycling, and functional resilience. Bridging these gaps requires integrating community profiling with microbial isolation, re-inoculation, gnotobiotic models, synthetic communities, and functional assays to validate microbial interactions and develop predictable, field-reproducible strategies for crop resilience.
